# A multimodal dataset of harmful simulated behaviours in high-risk clinical settings using radar

**DOI:** 10.1038/s41597-026-06703-8

**Published:** 2026-03-13

**Authors:** Benjamin Tilbury, Miguel Arevalillo-Herráez, Naeem Ramzan

**Affiliations:** 1 School of Computing, Engineering and Physical Sciences, University of the West of Scotland, Paisley, UK; 2 Departament d’Informàtica, Universitat de Valéncia, Burjassot, Valencia 46100 Spain; 3 Valencian Graduate School and Research Network of Artificial Intelligence (ValgrAI), Valencia, Spain

**Keywords:** Risk factors, Quality of life

## Abstract

We present a new dataset comprising radar, Electrocardiography (ECG), respiration, and inertial measurement signal recordings from 23 individuals while performing a series of simulated harmful behaviors. This dataset covers a range of actions across various levels of agitation and is especially well-suited for conducting research in health monitoring within high-risk clinical settings, such as inpatient psychiatric units. The dataset’s design prioritizes unrestricted, naturalistic behavior capture, providing valuable insights into real-world scenarios and supporting a wide range of applications. Although the dataset was initially designed for patient monitoring, the provided ECG and respiration recording extend the potential uses of the data to localization and non-contact vital sign measurement.

## Background & Summary

Remote human activity detection encompasses technologies and methodologies aimed at identifying and classifying physical actions or behaviors of individuals or groups without direct contact or physical intervention. The applications of remote human activity detection are vast, including security surveillance^1^, elderly care^2^, smart home automation^3^ and sports analysis^4^. In healthcare, where patient well-being is of paramount concern, the ability to comprehensively monitor activities holds profound implications for improving patient care, safety, and overall quality of life. Traditional activity monitoring methods, reliant on manual observation or wearable sensors, are often intrusive, labor-intensive, and may compromise patient dignity and privacy. Moreover, they may not be suitable for environments where patients are at an elevated risk of self-harm, necessitating constant supervision^5–8^.

Radar technology, specifically Ultra-Wideband (UWB) and micro-Doppler radar systems, have been extensively explored for detecting human activities through walls or in obscured conditions, demonstrating high potential for security, rescue operations, and activity analysis applications. Studies have shown that UWB radar can accurately classify human activities such as standing, walking, sitting, and lying with high precision by analysing the reflected signals from human movements^9^. Similarly, micro-Doppler signatures obtained from low-cost radar systems have been successfully used for outdoor human activity detection, achieving classification accuracies up to 100% in distinguishing different types of human motions^10^. As a related area of study, there also exists a substantial body of research investigating the utilization of radar technology for fall detection^11,12^.

In secure clinical health settings, such as psychiatric wards, intensive care units, or facilities caring for patients with cognitive disorders, the need for vigilant and continuous monitoring is particularly pronounced. These settings are characterized by a high degree of patient vulnerability, where lapses in supervision can have dire consequences. Staff-intensive monitoring, while essential, can be resource-intensive and may not provide the real-time insights needed to preemptively intervene in potentially harmful situations. Radar-based wireless activity detection offers a non-invasive and unobtrusive solution to these challenges. By harnessing the power of radio waves, radar technology can accurately and discreetly monitor the movements and activities of individuals in real-time, providing an invaluable layer of security and safety.

In this field, the generation of new models and methods is supported by the existence of labeled databases that are readily available and serve as both training data and reliable ground truths during development. These databases can be used to generate and test feature extraction algorithms and classification methods on the data provided. In addition, they also ease the validation and refinement of algorithms, as developers can iteratively compare their outputs against the known truths and iteratively refine their approaches. This motivation has spurred significant efforts towards the creation of multiple radar-based datasets, e.g.^13–18^. However, they present various limitations that make them ill-suited to address the specific needs of healthcare and secure clinical settings. First, they are very rigid in the participant’s representation of each activity, making them less conducive to transfer learning on complicated realistic, and less predictable movements. Second, they omit factors contributing to signal noise or degradation (movement, variable subject distances, etc.), precluding the development of holistic solutions that can be readily applied in practice. Third, they overlook behaviors associated with potentially harmful situations.

This paper introduces a novel dataset designed to represent real-world scenarios in high-risk clinical settings. The dataset offers recordings of 23 individuals while performing 12 different activities, some of which represent typical harmful behaviors. This dataset holds particular significance for the development of new methods in health monitoring within environments such as inpatient psychiatric units, covering actions across various levels of agitation. Unlike the datasets described above, we have synchronously captured additional data in the form of vital signs and motion-related measures. In particular, we have recorded ECG, respiration, and inertial measurement (gyroscope, accelerometer). These signals extend the range of applications of the dataset presented beyond activity detection. While there are other datasets specifically designed for vital sign detection in medical settings, e.g.^19,20^, they are restricted to static situations, generally seated positions, and do not include active scenarios. To our knowledge, this is the first dataset in the literature where activities, vital signs, and motion information have been simultaneously considered.

## Methods

### Informed consent

Ethical approval was obtained from the ethics committee of School of Computing, Engineering and Physical Sciences at University of the West of Scotland under project ID 18257. Because the study’s experimental protocol involved the continuous acquisition of physiologically sensitive real-time data, such as heart rate, respiration, and blood-oxygen saturation signals, it was mandatory to obtain explicit informed consent from every participant, including their consent for data sharing. Prior to any data recording, each prospective participant was presented with a detailed information sheet that outlined the purpose of the research, the nature and duration of the signal-monitoring procedures, potential risks and discomforts associated with wearing the sensor array, data-handling practices, and the safeguards in place to protect personal privacy and anonymize recorded traces. Participants were given the opportunity to ask questions, and withdraw at any point without prejudice. Only after their full understanding was confirmed through a short verbal debrief and a written comprehension checklist did they sign the consent form, thereby authorizing the collection, storage, and future analysis of their data. The ethics committee also approved the open publication of these data for research purposes.

### Recorded signals

The HOPE-R dataset aims to overcome the limitations of existing radar datasets. It integrates radar data alongside activity classes, ECG, respiratory, accelerometer, and gyroscopic signals, supporting the development of methods for activity detection, vital sign detection, and person localization tasks.

A XeThru X4M03 Impulse-Radio Ultra-Wideband (IR-UWB) radar device was used for the collection of radar data. The device samples received energy from transmitted radio frequency pulses in discretised range bins, resulting in a radio frequency (RF) frame. Using an in-built down converter the RF frames are converted into a complex baseband frame. We used a sampling rate of 300 frames per-second over 62 range bins. Bins start at a distance of ~0.386m (discarding smaller range bins that contain direct path clutter), and end at a distance of ~3.54m, (discarding larger range bins that are beyond the bounds of the testing area). The radar device was connected to a Windows 10 desktop PC (PC-A) that triggered it to start and stop and stored each frame received by the device. At the beginning of each experiment, the PC-A’s clock was manually resynchronized using w32tm /resync. Two UNIX-epoch timestamps at millisecond precision were taken when the radar device started and stopped at the beginning and end of each sequence, respectively. Frame rate is assumed to be consistent, and after the session ends, each received frame is assigned a timestamp uniformly interpolated between the start and stop timestamps based on its received index.

ECG and respiration signals were gathered from participants over the course of the experiment, using a Shimmer3 portable ECG device. The device features a respiration mode that uses impedance pneumography to measure the changes in impedance across a subject’s chest during inhalation/exhalation. The device was secured around the participant’s abdomen for the duration of the experiment with electrode placement as follows:

- RL. This is the reference electrode and was attached to the top of the right leg.
- LL. Attached to the top of the left leg.
- LA. Standard V6 ECG position—on the left side of the chest, along the mid-axillary line at the sixth intercostal space.
- RA. symmetrically on the right side of the chest at the same anatomical level as LA.
- Vx. V1 ECG position: on the right side of the sternum, at the fourth intercostal space.

RA and LA are used for respiration, with RL as the reference point, allowing LL and Vx to function as standard ECG channels.Additionally, the Shimmer3 device captured auxiliary accelerometer and gyroscopic signals. All signals from the device were sampled at 512Hz. Data was saved directly into the device throughout the experiment while also transmitting the data to PC-A for visualisation to ensure that there were no problems with lead disconnections. The device features a real-time clock (RTC) providing each sample collected with a UNIX-epoch timestamp at millisecond precision. The RTC is automatically synchronized at the start of each experiment by docking with PC-A.

### Setting

**Fig. 1:**
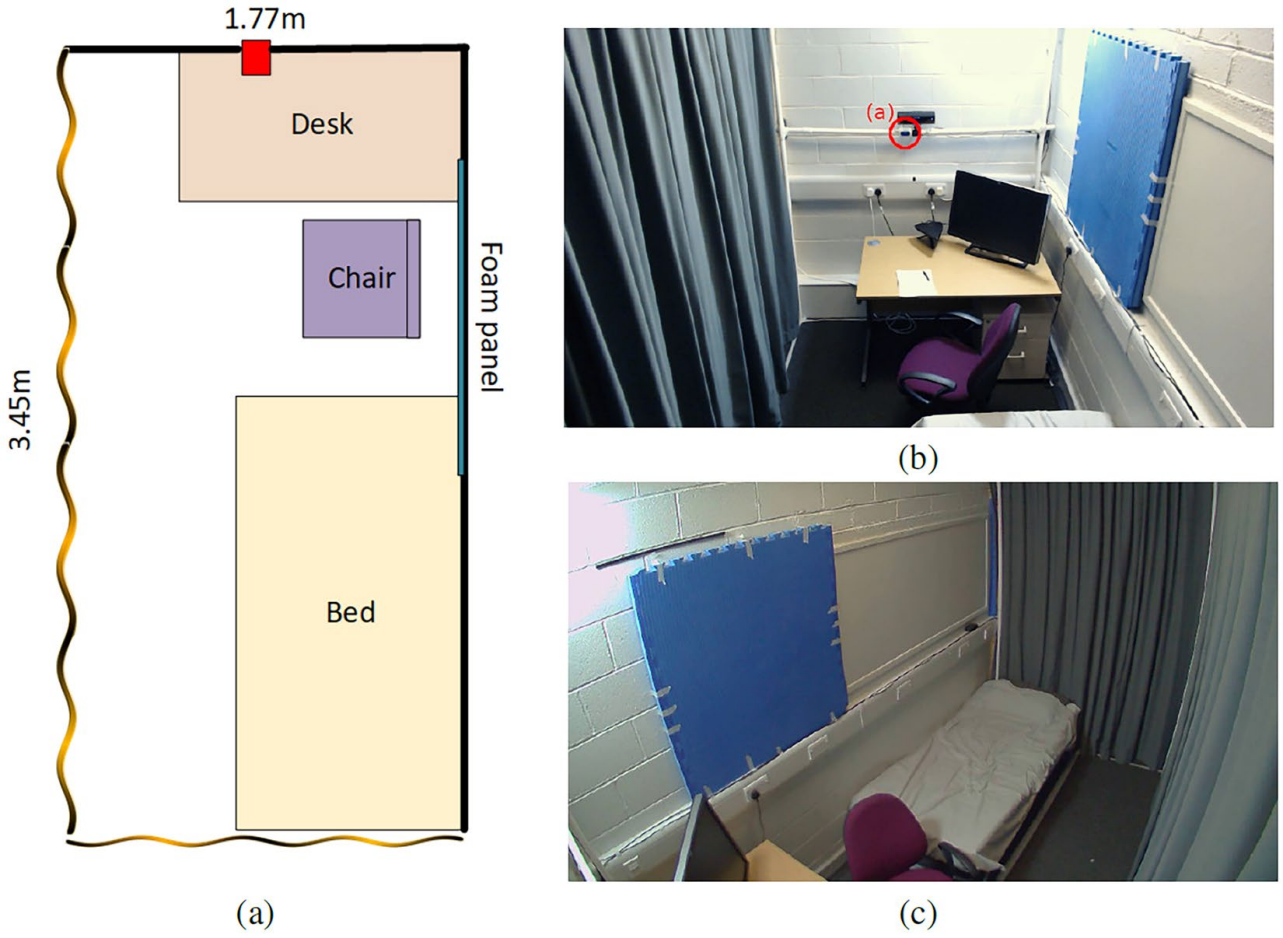
Floor plan of the experimental setting (**a**), along with two views of the testing area, showing the location of the XM403 Radar device (**b** and **c**).

A testing area was designed to simulate a minimal bedroom, typical of secure care units. The testing area was 3.45m long and 1.77m wide and contained a bed, desk, TV, and desk chair. The floor plan is shown in Fig. 1, along with two camera views. The red box in Fig. 1(a) represents the radar device, which is positioned on the back wall facing the length of the testing area. It is located slightly off-centre horizontally to minimize obstruction from the TV and lies directly in front of participants when they are sitting at the desk. Its vertical position is 1.4m to avoid obstruction by the TV and the desk chair when it is not in use. The wavy lines on the left-hand and bottom sides of 1(a) represent the curtains, while the solid, bold lines on the right-hand and top sides of the figure indicate sturdy brick walls.

### Activities considered

The list of activities is provided in Table 1. Participants were alone in the testing area for the duration of each sequence, except for the “Care Provider” activity class, during which an additional person entered the testing area and remained for 20 to 35 seconds before leaving.

**Table 1:** Activities classes chosen to reflect typical behaviors likely to occur in a real setting.

Class	Duration (s)	Position	Harmful
Standing	20 to 35	Variable	No
Sitting at Des k	20 to 35	Fixed	No
Sitting on Bed	20 to 35	Fixed	No
Sitting on Floor	20 to 35	Fixed	No
Pacing	20 to 35	Set Path	No
Watching TV	20 to 35	Fixed	No
Lying on Bed	20 to 35	Fixed	No
Care Provider	20 to 35	Variable	No
Agitated Sitting	20 to 35	Variable	Yes
Agitated Pacing	20 to 35	Set Path	Yes
Ligature behavior	20 to 35	Variable	Yes
Headbanging	20 to 35	Fixed	Yes

While most of the activity classes are self-explanatory, some warrant further explanation. The classes ‘Agitated Sitting’, ‘Agitated Pacing’, ‘Ligature Behavior’, and ‘Headbanging’ are designed to emulate harmful behaviors found in secure care units. Forms of harmful behavior are numerous, as are how they present. The behaviors chosen for inclusion in our dataset were based on three factors:

- The frequency of the behaviors in situ -as reported in interviews undertaken with staff at a secure care unit.
- The risk of serious harm or loss of life that the behavior may pose.
- The feasibility for our participants to simulate the behavior safely.

It should be noted that other forms of self-harm/harmful behaviors are more common under normal circumstances, with cutting representing the majority of cases of non-fatal self-harm in cases admitted to hospitals^21^. However, a core element of secure care units are the practices put in place to mitigate these behaviors from occurring (e.g. by removing sharp objects and ligature anchor points that could facilitate strangulation). The selected behaviors represent current concerns.

In addition to the harmful behaviors, the ‘Care Provider’ class was included to emulate the presence of a care provider attending to a resident in a secure care unit, though its usefulness is not restricted to this context and can be used to represent any situation in which two people are present in a room. For the ‘Care Provider’ class, participants were asked to stop their current activity and a researcher entered the testing area, moving around for 20 to 35 seconds before exiting.

Although visible expressions of agitation/distress/anxiety do not necessarily result in direct harm, they are an important indicator of a person’s mental state -the presentation and severity suggest an increase in the likelihood of directly harmful behavior. For the ‘Agitated Sitting’ and ‘Agitated Pacing’ activity classes, participants were asked to sit and pace respectively, while exaggerating agitation through physical movement. A demonstration of how to perform these two activities was shown to participants. However, to capture a variety of different physical manifestations of agitated behavior, participants were free to express agitation in a way they deemed suitable.

Because of the diligent removal of ligature anchor points in secure care units, anchored strangulation is uncommon. The “Ligature Behavior” activity class instead refers to and represents unanchored ligature strangulation. Participants simulated tying a ligature around their necks, followed by holding their hands and arms obscuring their necks for 20 to 35 seconds. This behavior is based on observations from the staff interviewed describing the common occurrence of residents finding or creating makeshift ligature, tying it tightly around their neck, and then guarding the area tightly with their hands and arms in an effort to prevent staff from getting to the ligature to cut it. To vary the data as realistically as we could, participants were instructed to perform the ligature behavior in the same position as their previous activity. As the order is randomised, this imparts randomness to the location of the ligature behavior class.

For the ‘Headbanging’ activity class, participants were asked to rhythmically hit their heads against a cushioned foam panel for 20 to 35 seconds. Because a wall is all that is required to perform this behavior, it is common and difficult to prevent in secure care units.

It should be noted that other activities such as human falls, which have a well–established impact on human health, could have further increased the dataset’s scope and attractiveness. Nevertheless, the collection of realistic fall data would have exposed participants to physical risk and raised ethical concerns, and was therefore not undertaken.

### Data collection

Data were collected from 23 unique participants. Given the inherent difficulties in recruiting for this type of study, it was not feasible to apply strict selection or stratification criteria. Consequently, participants were included on a voluntary basis as they became available. While demographic information such as age and gender was recorded, the sample size does not allow for a meaningful analysis of these factors, and they were therefore not included in the main analyses.

In total, 28 experimental sessions were conducted, as five participants completed an additional session on a different day. This design allowed us to distinguish between variations attributable to individual differences and those arising from session-specific external factors. Each experiment session consisted of 2 sequences inside the testing area, with each sequence lasting approximately 10 minutes. The first sequence corresponded to a standard scenario, while the second sequence considered an “after exercise” scenario, where participants engaged in moderate to intense exercise on a stationary bike for 8 minutes before data recording. The purpose of this second scenario was to introduce elevated heart rate, respiratory rate, and oxygen saturation values into the dataset providing more varied coverage of values that may be present in realistic situations.

All sequences started with the participant standing in the testing area by the bed, and ended with them exiting the testing area at which point recording is stopped. During each sequence, the participant acted out a set of 12 different activities in the testing area while ground truth vital signs were gathered via a Shimmer3 portable ECG device and O2Ring oximeter, in addition to the ground truth start and stop times for each activity. To avoid patterns in vital sign changes that may arise from a fixed-order set of activities and allow for a more even distribution of vital sign values across all activities, the order and duration of activities were randomised for each individual but kept consistent across the 2 sequences. Otherwise, no data would be captured with elevated vital signs for the activities toward the end of the sequence, as vital signs gradually return to normal after the exercise.

To facilitate the continuity of each sequence, a scheduling program was used to generate the randomised schedule of activities for the current experiment session and record the start and stop times of each activity that employed the following protocol for each sequence:

1. Record program launch time as the start timestamp for “Starting Time”.
2. Program waits the scheduled activity time.
3. Inform researcher next activity should start.
4. Researcher inputs confirmation and the program records the stop timestamp for the current activity.
5. Researcher informs participant of their next activity.
6. Researcher waits for visual confirmation that the participant has started the next activity.
7. Researcher inputs confirmation and the program records the start timestamp for the next activity
8. Repeat until participant has completed all activities.
9. Researcher asks participants to exit the testing area.
10. Researcher inputs confirmation and the program records the start time for “Stopping Time”.
11. Researcher waits until the participant has left the testing area.
12. Researcher inputs confirmation and the program records the stop time for “Stopping Time”.

The program was running on a separate Windows 10 PC (PC-B) and captured UNIX-epoch timestamps at nanosecond precision. Like PC-A, at the beginning of each experiment, the PC-B’s clock was manually resynchronized using w32tm /resync.

## Data Records

A summary of the signals recorded in this dataset^22^ can be found in Table 2. Each session is stored in an HDF5 file with the following structure:

**Table 2:** Summary of dataset signals.

Signal	Data Type	Units	Channels	Hertz
Xethru Radar	Complex 64-bit float	None (Ratio)	62	300
Oxygen saturation	32-bit integer	SpO2 level (%)	1	512
ECG	64-bit float	*mV*	4	512
Respiration	64-bit float	*mV*	1	512
Accelerometer	64-bit float	*m*/*s*^2^	3	512
Gyroscope	64-bit float	∘/*s*	3	512

The HDF5 file contains 2 HDF5 groups named STANDARD and AFTER_EXERCISE, corresponding to the two types of scenarios considered, as described in the Data Collection section. Each of these are divided into subgroups labeled as activities, shimmer and xethru.

The activities HDF5 group contains the following data with shared indices:

- name. Contains the names of the activities.
- start. Contains the timestamps signifying the start of the respective activity (nanoseconds since UNIX-epoch, nanosecond precision).
- stop. Contains the timestamps signifying the end of the respective activity (nanoseconds since UNIX-epoch, nanosecond precision).

The xethru HDF5 group contains the following data from the XeThru X4M03 device with shared indices:

- data. Contains the collected range-time-intensity matrix data in complex 32-bit floating point form. Columns are range bins ranging from ~0.386m to ~3.524m
- timestamps. Contains the timestamps corresponding to each entry in data (nanoseconds since UNIX-epoch, millisecond precision).

The o2ring HDF5 group contains the oxygen saturation levels captured by the O2Ring oximeter, along with the timestamps for each measurement.

The shimmer HDF5 group contains the following data from the Shimmer3 Portable ECG device with shared indices:

- LA_RA, LL_LA, LL_RA, and Vx_RA. Contain the raw ECG signals with 3 bipolar channels *LA* − *RA*, *LL* − *LA*, *LL* − *RA*, and the unipolar channel from *V*_1_.
- gyro_x, gyro_y, and gyro_z. Contain the *x*, *y*, and *z* gyroscope values. Oriented such that *x* is the transverse axis (*x* + left, *x* − right), *y* is the vertical axis (*y* + up, *y* − down), and *z* is the sagittal axis (*z* + anterior, *z* − posterior)
- accel_x, accel_y, and accel_z. Contain the *x*, *y*, and *z* accelerometer values. The accelerometer axes are aligned with those of the gyroscope.
- resp. Contains the impedance pneumography respiration signal.
- timestamps. Contains the timestamps corresponding to each entry in ecg, respiration, gyroscope, accelerometer (nanoseconds since UNIX-epoch, millisecond precision).

## Technical Validation

To ensure the technical quality of the captured data, the signals were plotted and visually inspected for validity. The original complex signal for each activity and subject in the standard scenario, originally captured at 300 Hz, was converted into a matrix of shape *n* × 62 of floating point values, where n is the number of samples and 62 is the dimension of the XeThru signal. All rows corresponding to the first and last 4 seconds (1 200 samples at each side of the signal) were discarded, to avoid potential effects caused by external factors such as the required keyboard and mouse interactions to indicate the change of activity. The logarithm in base 10 of the magnitude of each sample in the remaining complex signal was then computed. Then, 20 downsampled signals were produced, each starting at row *i* = 1…20 and taking one every 20 samples. Each of the downsampled signals was processed to extract windows of 60 samples each, applying a 90% overlapping (54 samples). Each window, of size 60 × 62, was then visualized as an image and labeled with the activity the user was engaged in when the original signal was recorded. Finally, we checked the plots for activity-related patterns that could be used to visually identify the activity.

**Fig. 2:**
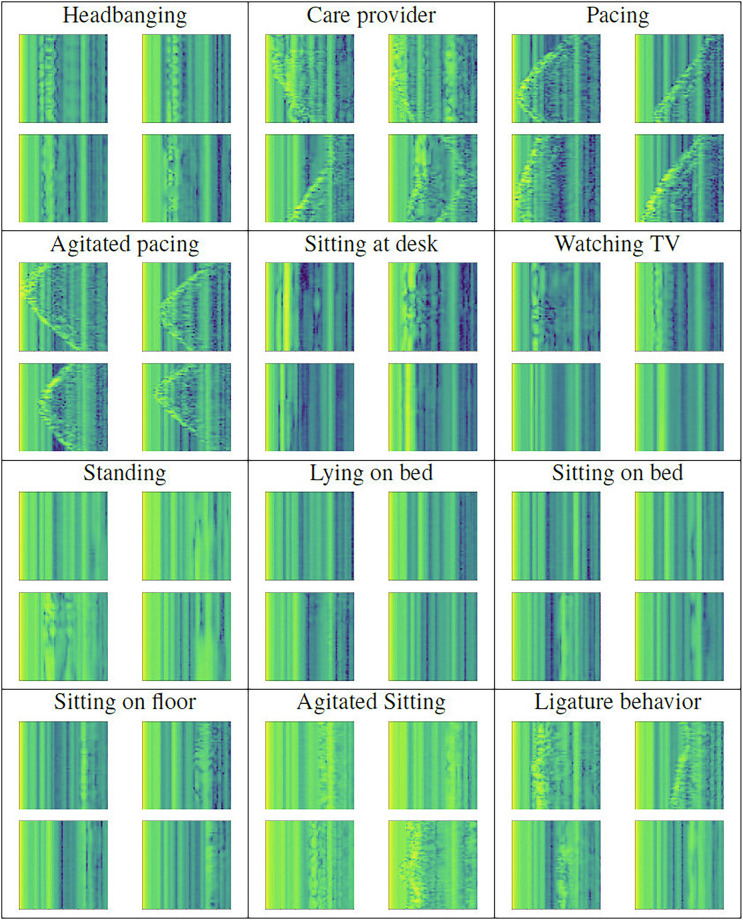
Illustrative samples for each activity. All samples for the same activity belong to different users.

Some images chosen at random for each activity are shown in Fig. 2 for each activity, taking care that all images for any given activity belong to different users. The color of pixel (*i*, *j*) corresponds to the application of a colormap which maps the lowest value to dark blue and the highest to bright yellow, using a scale between the two colors to represent intermediate values. The consistency of samples under the same activity group supports the reliability of the data captured. As can be observed, representations of the same activity across different users display consistent patterns. This finding supports the use of pattern-based classification techniques and machine-learning methods for reliable activity detection.

## Usage Notes

The dataset.zip file first need to be decompressed. The decompressed folder will contain one h5 file per session. To read the data for each session, we recommend using python, but h5 files can also be read from Matlab and R. Alternatively, the official HDFView viewer https://www.hdfgroup.org/downloads/hdfview/can be used to graphically inspect and export .h5 files. As an illustrative example, we provide below concise code demonstrating how the data corresponding to participant 0’s session can be inspected, as well as how the impedance pneumography respiration signal acquired by the Shimmer device in the standard scenario can be accessed using Python.


import h5pywith h5py.File("participant_0.h5", "r") as f:print(list(f.keys()))data = f["STANDARD/shimmer/resp"][:]


The dataset has been devised so that two different evaluations are possible. One first possibility is to analyse each activity individually, considering a correct result when the activity label is accurately predicted. A second possibility is to treat each sequence as a continuous series of activities. In this case, techniques and systems developed using our dataset could use the results across an entire sequence as a gold standard for its efficacy. A system can be poised for practical implementations if it is able to execute causally and in real-time on a complete sequence as if it were live video, accurately predicting each class while also correctly predicting no class for the transitional periods between activities.

In either case, we should note that although care was taken to ensure that class boundaries were started and stopped, we recommend discarding the first and last 4 seconds of each activity. We also recommend an incremental approach when evaluating techniques. Our dataset is expansive and very broad, encompassing a wide range of classes. Certain class subsets or collections when taken in isolation are similar to those in other existing datasets and allow for double-testing. For example, when developing a model for the prediction of heart rate and/or respiratory rate; it is recommended to start using only the —Sitting at Desk’ and —Watching TV’ classes, and also validate the results against other existing datasets that provide similar settings (close distance to the subject and little movement), such as the ones presented in^19,20^.

It should be noted that the behaviors examined in this study were simulated. Consequently, the observed outcomes may not fully reflect real behaviors, particularly with respect to ECG and respiratory measurements. This is especially relevant for activities such as ligature behavior, which could not be enacted for ethical and safety considerations.

## Data Availability

The dataset^22^ has been deposited to Zenodo^23^ and is available from 10.5281/zenodo.17565754.

## Code availability

No custom code was used.
